# Mutational Analysis of the Analgesic Peptide DrTx(1-42) Revealing a Functional Role of the Amino-Terminal Turn

**DOI:** 10.1371/journal.pone.0031830

**Published:** 2012-02-15

**Authors:** Ping Li, Shunyi Zhu

**Affiliations:** State Key Laboratory of Integrated Management of Pest Insects and Rodents, Institute of Zoology, Chinese Academy of Sciences, Beijing, People's Republic of China; Aston University, United Kingdom

## Abstract

**Background:**

DrTx(1-42) (a carboxyl-terminally truncated version of drosotoxin) is a potent and selective blocker of tetrodotoxin-resistant (TTX-R) Na^+^ channels in rat dorsal root ganglion neurons with analgesic activity. This purpose is to identify key amino acids which are responsible for both blocking and analgesic effects of DrTx(1-42).

**Methods:**

On the basis of previous study, we designed five mutants of DrTx(1-42) (delN, D8A, D8K, G9A, and G9R) in the amino-terminal turn (N-turn) region, a proposed functional region located in the amino-terminus of the molecule. All these mutants were expressed in *E.coli* and purified by RP-HPLC. Electrophysiological properties of these analogues were examined by whole-cell patch-clamp recordings and their antinociceptive effects were investigated by the formalin test and acetic acid induced writhing test.

**Results:**

All the mutants except for G9A possess a similar secondary structure to that of DrTx(1-42), as identified by circular dichroism analysis. Three mutants (delN, D8A and G9A) were found almost inactive to TTX-R Na^+^ channels, whereas D8K retains similar activity and G9R showed decreased potency when compared with the wild-type molecule. Consistent with the electrophysiological observations, D8K and G9R exhibited antinociceptive effects in the second phase (inflammatory pain) of the formalin test and the acetic acid induced writhing test, while delN, D8A and G9A lack such effects.

**Conclusions:**

Our results show that the N-turn is closely related to function of DrTx(1-42). The mutant (D8A) as a control peptide further reveals that a charged residue at site 8 of the N-terminus is important for channel blockade and analgesic activity. This study indicates that blocking of voltage-gated TTX-R Na^+^ channel in DRG neurons contributes to analgesic effect in rat inflammatory pain. Structural and functional data described here offers support for the development of novel analgesic drugs through targeting TTX-R Na^+^ channels.

## Introduction

Inflammatory pain, caused by tissue injury or inflammation, is a serious medical problem worldwide and especially difficult to treat [1]. Voltage-gated Na^+^ channel (Nav) blockers have been clinically validated as treatments for inflammatory pain. However, non-selective inhibitors of Navs generally have dose-limiting central nervous system and cardiovascular side effects, which prevent their use in long term therapy [2], [3]. Previous studies have showed that the deletion of TTX-R Nav genes or pharmacological inhibition of their functions can markedly reduce some inflammatory pains [4], [5], recent study also validated that antisense-mediated knockdown of Nav1.8 -TTX-R sodium channel generated inhibitory effects on Complete Freund's Adjuvant-Induced inflammatory pain in rat [6], supporting the importance of TTX-R sodium channels as new targets to develop therapeutic agents for inflammatory pain.

Navs are large transmembrane proteins that mediate the rising phase of the action potential in excitable cells. In mammals, there are nine Nav subtypes (Nav1.1-Nav1.9) identified, all having distinct tissue distributions and biophysical properties [7]. Based on their sensitivity to TTX, these nine Navs can be classified as either TTX-sensitive (TTX-S) (eg, Nav1.1-Nav1.4, Nav1.6 and Nav1.7) or TTX-R (eg, Nav1.5, Nav1.8 and Nav1.9) [8]. Two remarkable TTX-R channels , Nav1.8 and Nav1.9 are predominantly expressed in nociceptive neurons in the dorsal root ganglion [2].

Because of key roles of TTX-R Na^+^ channels in inflammatory pain sensation, it is extremely desirable to search for specific blocker of these channels as drug leads. Animal venoms have been proved to be a rich source of peptide toxins that function as modulators or blockers of Navs [9], [10]. However, the majority of these toxins were reported to only affect TTX-S Na^+^ channels, and the only one naturally-occurring blocker (Conotoxin mμ-SIIIA) selectively targeting mammalian TTX-R sodium channels was identified from Conus striatus, 3 µM mμ-SIIIA could almost completely inhibit TTX-R Na^+^ currents [11]. In addition, two conotoxins (μO-MrVIA and μO-MrVIB) were found to preferentially block mammalian TTX-R over TTX-S channels, and their respective IC_50_s of inhibition to TTX-R currents were 82.8 and 98 nM. Accordingly, μO-MrVIB reduced both inflammatory and neuropathic pain [5].

Recently, we reported an engineered chimeric peptide drosotoxin, which was achieved by using drosomycin (*Drosophila* antifungal defensin) to substitute the structural core of BmKITc, a weak depressant toxin acting on both insect and mammalian Na^+^ channels. Our data indicated that recombinant drosotoxin possessed strong potential to selectively block TTX-R Na^+^ currents in rat DRG neurons with a 50% inhibitory concentration (IC_50_) of 2.60±0.50 µM [12]. During production of drosotoxin, we unexpectedly achieved a C-terminally truncated drosotoxin DrTx(1-42), which also displayed high inhibitory effect on rat TTX-R Na^+^ channels with an IC_50_ value of 1.74±0.07 µM. Furthermore, we found that DrTx(1-42) was significantly able to reduce inflammatory pain in the formalin test [13].

Given promising therapeutic potential of DrTx(1-42), a logical next step is to understand its structure-function relationship for guiding further molecular design. Here we aim at previously proposed functional region of this peptide to highlight key amino acids sites essential for blockade as well as analgesic effect by using mutational experiments combined with electrophysiological and antinociceptive assays. Our results highlight the functional importance of a charged residue at the N-turn of DrTx(1-42).

## Materials and Methods

### Ethics statement

All experimental protocols were reviewed and approved by the Animal Care and Use Committee of Institute of Zoology, the Chinese Academy of Sciences. The institute does not issue a number to any animal study, but each study requires the permit to use animals from the ethical committee. The animal facility must be licensed by the experimental animal committee of Beijing, and all staff, fellows and students must receive appropriate training before performing animal studies.

### Materials

ATP, T4 DNA ligase and ExTaq polymerase were obtained from Takara (Dalian, China); Pfu polymerase and polynucleotide kinase were respectively purchased from newProbe (Beijing, China) and TOYOBO (Osaka, Japan); Glutathione Sepharose 4B beads was purchased from GE Healthcare (Shanghai, China); Fetal bovine serum (FBS) was purchased from Sijiqing Biotech Co. Ltd. (Hangzhou, China); Enterokinase (EK) was obtained from Sinobio Biotech Co. Ltd. (Shanghai, China); tetrodotoxin (TTX), trypsin (type III), collagenase (IA), trypsin inhibitor (type II-S), Dulbecco' s modified Eagle' s medium (DMEM), tetraethylammonium chloride (TEA-Cl), D-glucose, 4-(2-hydroxyethyl)-1-piperazineethanesulfonic acid (HEPES), CsF, CsOH, CsCl, NaCl, KCl, MgCl_2_, CaCl_2_, and NaOH were purchased from Sigma - Aldrich (Shanghai, China). All these reagents were of analytical grade.

### Animals

All animals were purchased from Experimental Animal Center, Academy of Military Medical Sciences. Animals were housed in a temperature controlled room (22–25°C) with water and food available ad libitum, and on a 12 h light/dark cycle.

### Site-directed mutagenesis

All primers used in this study were synthesized by SBS Genetech (Beijing, China) (Table 1). Inverse PCR, as previously described [14], was used to generate DrTx(1-42) (GenBank accession number GQ422754) and its mutants by using the recombinant plasmid pGEX-6P-1-drosotoxin and pGEX-6P-1-DrTx(1-42) as templates [12]. Phosphorylation of the 5′-end of primers was performed using polynucleotide kinase and ATP. PCR reaction conditions were as follows: 30 cycles of 45 s at 94°C and 8 min at 68°C with ExTaq DNA polymerase. Subsequently, linear PCR products were circularized by T4 DNA ligase after end polishing using Pfu polymerase. Circularized products were transformed into *E. coli* DH5α competent cells. Positive clones were confirmed by DNA sequencing.

**Table 1:** Primers used in this study.

DrTx(1-42) and Mutants	Primer Name	Sequence
DrTx(1-42)	DrTx(1-42)-FP	5′-TAAGTCGACTCGAGCGGCCGCATCGTG-3′
	DrTx(1-42)-RP	5′-CTTCAGACTGGGGCTGCAGTGGCCACT-3′
D8K	D8K-FP	5′-AAGGGATGCTACAAGGGTCCCTGTGCCGTC-3′
	D8K-RP	5′-ACTTCTTCCGGACAGGCCGTCCTTGTCATC-3′
G9R	G9R-FP	5′-AGATGCTACAAGGGTCCCTGTGCCGTCTG-3′
	G9R-RP	5′-GTCACTTCTTCCGGACAGGCCGTCCTTG-3′
D8A	D8A-FP	5′-GCCGGATGCTACAAGGGTCCCTGTGCCGTC-3′
	D8A-RP	5′-ACTTCTTCCGGACAGGCCGTCCTTGTCATC-3′
G9A	G9A-FP	5′-GCCTGCTACAAGGGTCCCTGTGCCGTCTG-3′
	G9A-RP	5′-GTCACTTCTTCCGGACAGGCCGTCCTTG-3′
del N	delN-FP	5′-TACAAGGGTCCCTGTGCCGTCTGGGAC-3′
	delN-RP	5′-TCTTCCGGACAGGCCGTCCTTGTCATC-3′

Note: Mutated nucleotides are underlined once. All primers listed here were synthesized by SBS Genetech (Beijing, China).

### Expression, purification and identification of recombinant products

Expression and purification of DrTx(1-42) and its mutants were performed according to the methods previously described [15]. In brief, the expression of recombinant DrTx(1-42) and its mutants in *E. coli* BL21 (DE3) was induced by 0.5 mM isopropyl- beta-D-thiogalactopyranoside (IPTG) and fusion protein was acquired in supernatant after sonication, followed by affinity chromatography with Glutathione–Sepharose 4B beads. Then the fusion protein was cleaved with enterokinase at 4°C overnight. The released protein samples were separated by RP-HPLC on C18 column (Agilent Zorbax 300SB, 4.6 mm×150 mm, 5 µm) using a linear gradient of 0–60% acetonitrile in 0.1% trifluoroacetic acid (TFA) in water (v/v) within 40 min with a flow rate of 1 ml/min, with detection at 225 nm. Peptides were eluted as major peaks at 28–30% acetonitrile. Molecular weights (MWs) of purified products were determined by matrix-assisted laser desorption ionization time-of-flight (MALDI-TOF) mass spectra on a Kratos PC Axima CFR plus (Shimadzu Co. Ltd, Kyoto).

### Circular dichroism analysis

The secondary structures of purified DrTx(1-42) and its mutants were measured by circular dichroism (CD) spectroscopy. All the peptides were dissolved in water at a concentration of 0.3 mg/ml. Measurements were carried out in the UV range of 260–190 nm at 25°C using a quartz cell of 1.0 mm in thickness on a JASCO J-720 spectropolarimeter. For each peptide, spectra were collected from 3 separate recordings and averaged at 0.5 nm intervals with a scan rate of 50 nm/min.

### Isolation of DRG neurons

DRG neurons were obtained as described in Xiao *et al.* [16]. Briefly, Adult male Sprague - Dawley rats (4 weeks old) were killed by decapitation without anesthetization, the DRGs were removed quickly from the spinal cord, and after having been cut into as small tissues as possible, they were transferred into DMEM containing trypsin (0.15 mg/ml, type III), collagenase (0.4 mg/ml, type IA) to incubate at 34°C for 20–30 min. Trypsin inhibitor (0.25 mg/ml, type II-S) was added to terminate the reaction. After centrifugation and resuspension, cells were plated on 13 mm diameter glass cover slips which had been precoated with poly-L-lysine (100 µg/ml, Sigma) and kept at 37°C in a humidified incubator gassed with 5% CO_2_ in air for 30–60 min. And then 2 ml DMEM containing 10% heat-inactivated fetal calf serum and 100 u/ml penicillin–streptomycin were added into the dish. Cells were cultured for 2–4 h before patch-clamping.

### Whole-cell Patch-Clamp Recordings

The Whole-cell configuration of patch-clamp technique was used to record Na^+^ currents in DRG neurons [16]. For Na^+^ current recordings, the pipette internal solution contained (in mM): CsF 135, NaCl 10 and HEPES 5, adjusted to pH 7.2 with CsOH. The external bathing solution contained (in mM): NaCl 30, KCl 5, CsCl 5, D-glucose 25, MgCl_2_ 1, CaCl_2_ 1.8, HEPES 5 and TEA-Cl 90, pH 7.4 with NaOH. TTX-R Na^+^ currents (TTX-R *I*_Na_) were recorded from small DRG neurons (≤25 µm diameter) in the presence of 300 nM TTX. Micropipettes were pulled from borosilicate glass capillary tubing by P-97(Sutter Instrument Co., Novato, CA, USA), the resistances of micropipettes were 3–6 MΩ after filled with internal solution. Na^+^ current traces were evoked by 50 ms depolarization from a holding potential of −80 mV to −10 mV. Currents were measured using an AxoClamp 2B amplifier (Axon Inc.) and DIGIDATA 1322A (Axon Inc.). Series resistance was routinely compensated by 70–80%. Capacitive and leakage currents were digitally subtracted by using a P/4 pulse protocol. Pulse stimulation and data acquisition were controlled by Clampex 9.0 software (Axon Inc.). Data are expressed as mean ± standard errors (SEM), the IC_50_ of mutant was calculated according to the Hill equation: I = I_max_/[1+(IC_50_/[A])^H^], where I_max_ is the control current to the peak, I is the remained current after adding polypeptide, A is concentration , and H is the Hill coefficient.

### Formalin Test

Healthy male ICR (imprinting control region) mice, weighing 18–22 g, were used in this study. The procedure used here was similar to the method described by Gomes et al [17]. Briefly, 20 µL of 2.5% formalin (v/v in distilled water) was injected into the ventral surface of the mice's right hind paws. Immediately after formalin injection, animals were individually placed in a glass observation chamber with a transparent floor. The summation of time (in seconds) that animals spent licking the injected paws was recorded from 0 to 5 min (neurogenic phase, corresponding to a direct chemical stimulation of nociceptors) and from 15 to 30 min (inflammatory phase, involving release of inflammatory mediators) after injection of formalin. Mice were pre-treated (i.v.) with peptides (0.1, 0.5 and 1 mg/kg) or vehicle (saline), 30 min before injection of formalin. In the positive control group, animals were pre-treated with indomethacin (10 mg/kg, i.v.). Results are expressed as mean ± standard errors (SEM) ,and analyzed as follows: Student Newman-Keuls post hoc test, one-way analysis of variance (ANOVA). A value of p<0.05 was considered to be statistically significant.

### Acetic acid induced writhing test

Healthy male ICR (imprinting control region) mice, weighing 18–22 g, were used in this study. Writhing test was performed as visceral inflammatory pain model [18]. Abdominal writhing is a model of visceral pain and nociceptive stimulus was induced with 0.6% acetic acid solution intraperitoneally injected to mice (0.1 ml/10 g of body weight). The number of writhes, which consists of abdominal muscle contractions and hind paw extension, were recorded for 15 min starting 5 min after i.p. injection. Each experimental group was formed by 8 mice. Animals were pre-treated (i.v.) with peptides (0.1, 0.5 and 1 mg/kg) or vehicle (saline), 30 min before administration of acetic acid. In the positive control groups, animals were pre-treated with indomethacin (10 mg/kg, i.v.). Results are expressed as mean ± standard errors (SEM) ,and statistically analyzed as same as above formalin test.

## Results

### Design strategy of mutation

**Figure 1:**
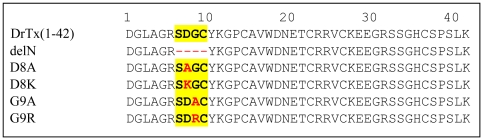
Sequence alignment of DrTx(1-42) and its mutants. The N-turn region derived from BmKITc is shadowed in yellow. Mutated sites are indicated in red.

Our previous reported data indicated that both drosotoxin and DrTx(1-42) were potent blocker to TTX-R channels, and both encompass the N-turn sequence grafted from BmKITc, accordingly we assume that this sequence motif could play a crucial role in the interaction with TTX-R Na^+^ channels. To test this assumption, firstly we designed an N-turn-deleted mutant (named delN) and found that delN almost had no inhibitory effects on TTX-R *I*_Na_ at 30 µM. Because of its similar structural feature to DrTx(1-42), the absence of activity provides direct evidence in favor of the importance of the N-turn in the interaction with TTX-R Na^+^ channels. Subsequently to identify which residues in the N-turn are possibly involved in the interaction with the channels, we further carried out a series of site-directed mutagenesis of the N-turn (D8A, D8K, G9A and G9R) (**Fig. 1**) and evaluated their electrophysiological and antinociceptive function.

### Recombinant production of DrTx(1-42) and its mutants

**Figure 2:**
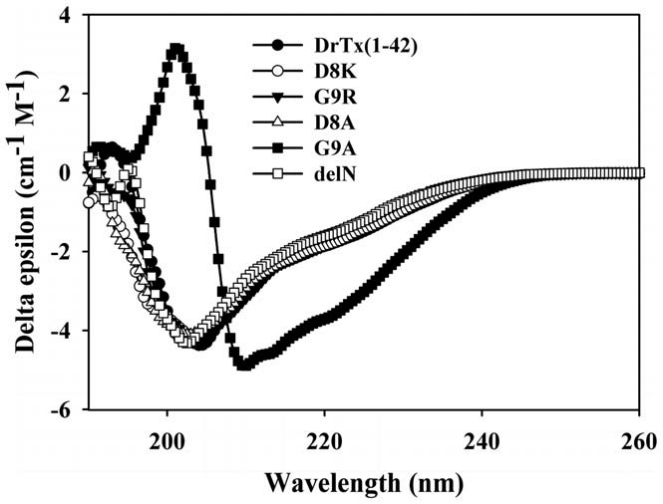
Circular dichroism spectra of DrTx(1-42) and its mutants. The measurement was carried out in the UV range of 260–190 nm at 25°C in water on a JASCO J-720 spectropolarimeter by averaging three scans. The protein concentration was 0.3 mg/ml.

Expression, purification and identification of DrTx(1-42) and its mutants were presented in **Fig. S1, Fig. S2, Fig. S3**). The MWs of all HPLC-purified mutants were confirmed by MALDI-TOF, which show that the MWs of all the recombinant products are close to their theoretical MWs (**Table S1** and **Fig. S4**, provided as electronic supplementary material). The CD spectra of these products were shown in **Fig. 2**, from which it can be seen that except for G9A, the spectra of all other mutants were found to be similar to that of DrTx(1-42), indicating no major secondary structure alterations in these mutants. However, the CD spectrum of G9A indicates that it is more helical compared to the other proteins.

### TTX-R Na^+^ channel blocking activities of DrTx(1-42) mutants

**Figure 3:**
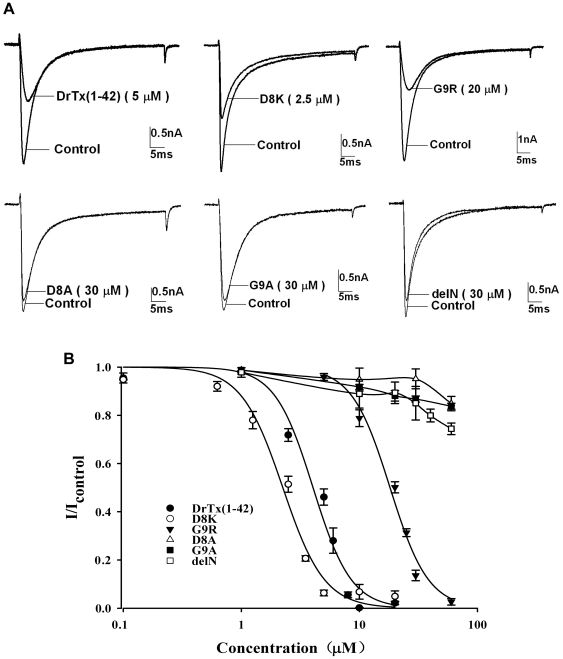
Effects of DrTx(1-42) and its mutants on TTX-R Na^+^ currents in rat DRG neurons (≤25 microm diameter). *A* Representative current traces of TTX-R Na^+^ currents blocked by DrTx(1-42) and its mutants. TTX-R *I*_Na_ was evoked by 50 ms depolarization from a holding potential of −80 mV to −10 mV. *B* The concentration-dependent inhibition of TTX-R Na^+^ channels by DrTx(1-42) and its mutants. Each point is the mean ± SEM for n = 5–8. These data points (DrTx(1-42), D8K and G9R) were fitted according to the Hill equation, the yielded IC_50_ values as follows: DrTx(1-42): 4.05±0.35 µM; D8K: 2.25±0.15 µM; G9R: 18.19±1.02 µM. NOTE-Recombinant DrTx(1-42) prepared in this study possesses comparable potency on TTX-R *I*_Na_ with that previously reported (Zhu *et al.*, 2011).

**Figure 4:**
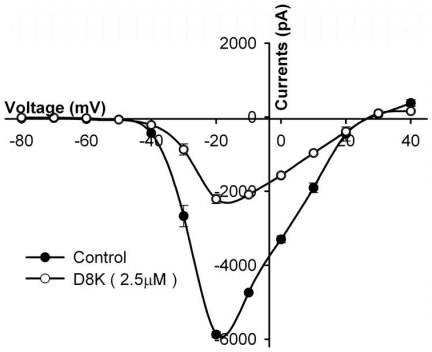
The current - voltage (I–V) relationship of D8K on TTX-R currents. **I–V** relationships of TTX-R currents before (filled circles) and after (open circles) the treatment of 2.5 µM D8K, respectively. Cells were held at −80 mV, and TTX-R currents were elicited by 50 ms depolarizing steps to various potentials ranging from −80 mV to +40 mV in 10 mV increments. Only D8K is presented here for example.

The blocking activities of these mutants were tested on TTX-R Na^+^ channels. The representative current traces of these mutants were showed in **Fig. 3A**. The mutant D8K significantly inhibited TTX-R *I*_Na_ at 2.5 µM concentration (n = 8), G9R inhibited about 60% TTX-R *I*_Na_ at 20 µM concentration (n = 5), whereas other mutants (D8A, G9A and delN) even at the concentration up to 30 µM had almost no effect on TTX-R *I*_Na_ (n = 8). Furthermore we also found blockade to TTX-R channels with DrTx(1-42), D8K and G9R was reversible since TTX-R *I*_Na_ could be recovered after washout (data shown in Fig. S5). As illustrated in **Fig. 3B**, when compared with DrTx(1-42), the mutant D8K showed almost the same activity (IC_50_ = 2.25±0.15 µM, n = 8), while G9R showed decreased activity (IC_50_ = 18.19±1.02 µM, n = 5). Effect of 2.5 µM D8K on the current - voltage (I–V) curves of TTX-R currents are illustrated in **Fig. 4**. From the curve, the inhibition to TTX-R currents was observed by 2.5 µM D8K, and the current inhibition was not associated with a change of the shape of the I–V relationship. Furthermore no shift in the membrane reversal potential of the TTX-R channels was observed, implying that D8K does not change the ion selectivity of the channels. The I–V curve indicates that inhibition effect of D8K on TTX-R sodium currents is the same as that of DrTx(1-42) and DrTx [12], [13].

### Analgesic effects of the DrTx(1-42) mutants in the formalin test

**Figure 5:**
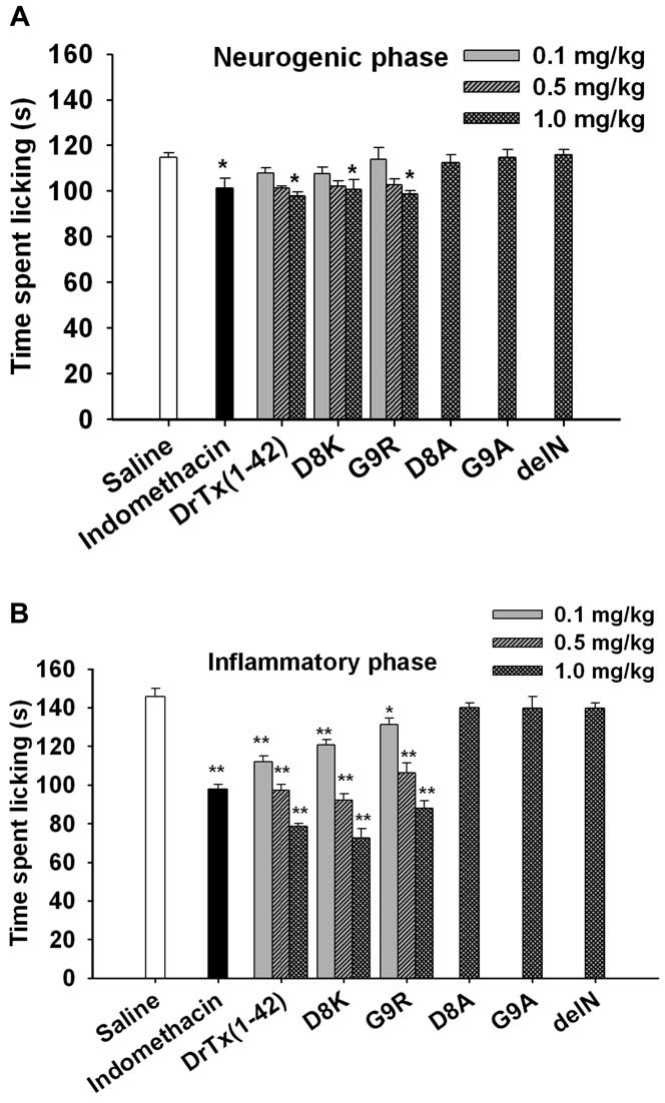
Effects of DTX(1-42) and its mutants on the time spent licking during the neurogenic (A) and inflammatory (B) phases of the formalin test in mice. The y-axis represents the time spent licking in sec during neurogenic and inflammatory phases. Data are reported as mean ± SEM for n = 8 per group. One-way ANOVA followed by Student Newman Keuls post hoc test. *: P<0.05, **: P<0.01 compared to vehicle-treated group (saline).

Given blockers targeting TTX-R Na^+^ channels have been characterized as effective antinociceptive agents [19], we evaluated the relationship of channel-blockade and antinociceptive activity of the DrTx(1-42) mutants in the formalin test in mice. During the first phase (neurogenic phase) of the formalin test, the administration of D8K and G9R only with maximal dose (1 mg/kg each) caused slight reduction in licking time of mice compared to the saline control (p<0.05) (**Fig. 5A**). In the second phase (inflammatory phase) of the formalin test, we found that the treatment by D8K and G9R (0.1, 0.5 and 1 mg/kg) significantly reduced licking time (p<0.01). The administration of mutants D8K and G9R with maximal dose (1 mg/kg) resulted in a significant inhibition of licking time by 50.17% and 39.62% respectively, which is comparable with positive control indomethacin (10 mg/kg). Indomethacin is a well-known non-steroidal anti-inflammatory drug acting through the inhibition of the inflammatory mediators of the acute phase of inflammation and is used frequently worldwide for the alleviation of pain despite their capacity to cause adverse gastrointestinal side effects [20]. In contrast, administration of mutants D8A, G9A and delN with maximal dose (1 mg/kg) had no significant pharmacological influence on licking time (**Fig. 5B**).

### Analgesic effects of the DrTx(1-42) mutants in the writhing tes*t*

**Figure 6:**
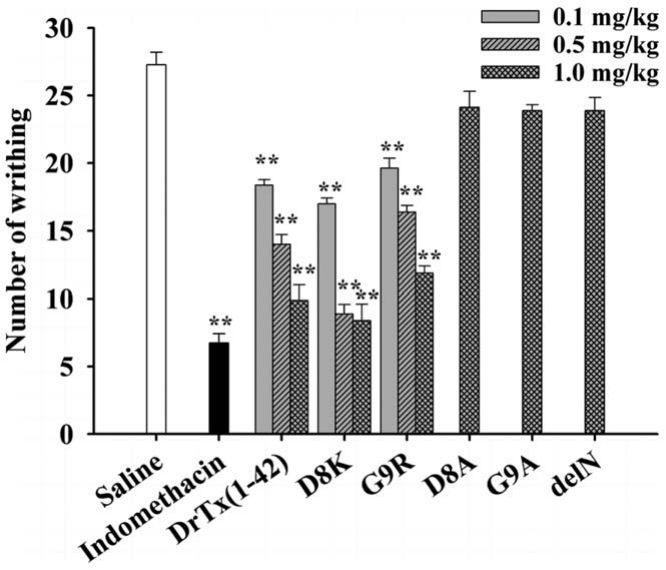
Effects of DrTx(1-42) and its mutants on acetic acid-induced writhing test in mice. Data are reported as mean ± SEM for n = 8 per group. One-way ANOVA followed by Student Newman Keuls post hoc test. *: P<0.05; **: P<0.01 compared to vehicle-treated group (saline).

Subsequently, we used another pain model by intraperitoneal injection of acetic acid that causes visceral inflammatory pain due to liberation of several mediators, such as histamine, serotonin, bradykinin, cytokines, and eicosanoids [21], [22]. These inflammatory mediators are able to increase vascular permeability, as well as to reduce the threshold of the nociception and to stimulate the nervous terminal of nociceptive fibers [23].The administration of mutants D8K and G9R at three doses (0.1, 0.5 and 1 mg/kg) caused a significant reduction in the writhing number induced by acetic acid in a dose-dependant manner (**Fig. 6**), in which both with maximal dose (1 mg/kg) resulted in the inhibition of writhing by 69.27% and 56.42%, respectively, comparable with positive control indomethacin (10 mg/kg). In contrast, the administration of mutants D8A, G9A and delN with maximal dose (1 mg/kg), no significant analgesic effects were found (**Fig. 6**). Animal behavioral observations indicate that D8K and G9R have obvious antinociceptive effects during the inflammatory phase of the formalin and the acetic acid induced visceral inflammatory pain model and thus support that DrTx(1-42), D8K and G9R are ideal candidates of analgesics. Blocking effects of these mutants on TTX-R *I*_Na_ in rat DRG neurons are consistent with their antinociceptive effects in animal tests obtained (Table 2). Their consistency further demonstrates that the inhibition of TTX-R Na^+^ channels will be a promising strategy to develop new analgesics.

**Table 2:** Comparison of channel blockade and antinociceptive effects of DrTx(1-42) and its mutants.

Groups	IC_50_ (µM)	Formalin test		Writhing test Inhibition (%)
		Neurogenic phase Inhibition (%)	Inflammatory phase Inhibition (%)	
DrTx(1-42)	4.05±0.35	14.72±1.60	46.14±1.15	63.76±4.24
D8K	2.25±0.15	12.21±3.13	50.70±3.18	69.27±4.49
G9R	18.19±1.02	13.85±1.27	39.62±2.71	56.42±1.89
D8A	>100	3.60±2.63	3.95±1.76	11.47±4.36
G9A	>100	2.41±2.73	4.14±3.28	12.39±1.62
delN	>100	0.11±1.48	4.04±1.95	12.39±3.57

Note: IC_50_ means the concentration of 50% inhibition on TTX-R Na^+^ currents in rat DRG neurons Antinociceptive effects of DrTx(1-42) and its mutants were obtained by using 1 mg/kg of dose (i.v.) compared to vehicle-treated group (saline). Data are shown as mean ± S.E.

## Discussion

DrTx(1-42) is a potent and selective blocker of TTX-R Na^+^ channels in DRG neurons with analgesic activity [13]. The present study is to identify key amino acids which are responsible for both blocking and analgesic effects of DrTx(1-42). On the basis of previous study [13], we firstly constructed a delN mutant, which had a similar CD spectrum to that of DrTx(1-42), but lose inhibitory effect on TTX-R *I*_Na_ and analgesic activity. These observations indicate that the N-turn is important for the function of DrTx(1-42).

Subsequently, we further carried out a series of site-directed mutagenesis of the N-turn (D8A, D8K, G9A and G9R) and found when Asp8 in the N-turn was substituted with an Ala, the blockade activity of this peptide on TTX-R Na^+^ channel nearly completely lose. However, when this acidic residue was reversed into a Lys, the mutant D8K retained almost the same blockade activity compared with DrTx(1-42). Based on structural similarity between these two mutants and DrTx(1-42), it appears that a charged group at site 8 (a positive or negative charge) is crucial for blocking of TTX-R Na^+^ channels. Furthermore it also indicates that D8 binds to TTX-R Na^+^ channels by a non-electrostatic interaction mode. Given these observations, it is reasonable to infer that a hydrophilic charged group on the side chain of site 8 possibly aids its positioning to favor an orientation in the solvent. Romeo *et al* reported that the mutant V6K at N-terminal segment of discrepin increased the blocking effect of potassium channel [24]. Our lab has previously found that the mutants D1A, R6A and K8A of drosomycin markedly decreased the antifungal activity of drosomycin [25]. Although these three peptides are by no means comparable in mechanism of action, it is interesting to observe a strategic commonality which hydrophilic charged group on the side chain at N-terminal is crucial to their activities. From the results of CD spectra, substitution of Gly9 by an Ala resulted in generating a more helical structure, which is different from the other peptides. Compared to the other peptide, the structure of G9A is more helical, but it completely abolished inhibition activity. But for the mutant G9R without structural change, an even lower activity was observed. It indicates that the structural change in G9A has a larger effect on function than just substituting the residue without structural change. Structure and activity analysis of G9A validates that more tight structure is not often associated with higher activity, as previously observed in an insect defensin [26].

### Conclusion

This study indicates that blocking of voltage-gated TTX-R Na^+^ channel in DRG neurons contributes to analgesic effect in rat inflammatory pain. In addition, it is clear that a charged residue in the N-turn of DrTx(1-42) could play a key role in such blockade. Structural and functional analysis of the analgesic DrTx(1-42) provides molecular basis for further design of new analgesics for pain treatment.

## Supporting Information

Figure S1 Expression vector map showing the position of DrTx(1-42).
(DOC)

Figure S2 Inverse PCR-mediated strategy to construct expression vectors, in which pGEX-6P-1-drosotoxin was initially used as template to generate the DrTx(1-42) expression vector which was further used to construct all the five mutants, including delN (SDGC deleted), D8K, D8A, G9A, and G9R.
(DOC)

Figure S3 RP-HPLC showing the purification of DrTx(1-42). Elution was carried out using a linear gradient of 0–60% acetonitrile in 0.1% trifluoroacetic acid in water (v/v) within 40 min with a flow rate of 1 ml/min. The re-purification is provided in inset.
(DOC)

Figure S4 Determination of molecular weights of HPLC-purified DrTx(1-42) and its mutants by MALDI-TOF.
(DOC)

Figure S5 Time - effect curves of mutants on TTX-R sodium peak currents. The channels were depolarized to −10 mV from a holding potential of −80 mV. A. 8 µM DrTx(1-42); B. 5 µM D8K; C. 30 µM G9R.
(DOC)

Table S1 Molecular weights of DrTx(1-42) and its mutants.
(DOC)
