# Supplementary material for: Mutational Analysis of the Analgesic Peptide DrTx(1-42) Revealing a Functional Role of the Amino-Terminal Turn
Source: PLoS One. 2012 Feb 15;7(2):e31830. doi: 10.1371/journal.pone.0031830 (PMC3280213; doi:10.1371/journal.pone.0031830)
Supplement: Figure S1 — Expression vector map showing the position of DrTx(1-42). (DOC) [file pone.0031830.s001.doc]

**Figure S1**

EK

*Bam* HI-DDDDKDGLSGRSDGCYKGPCAVWDNETCRRVCKEEGRSSGHCSPSLK-end-*Sal* I


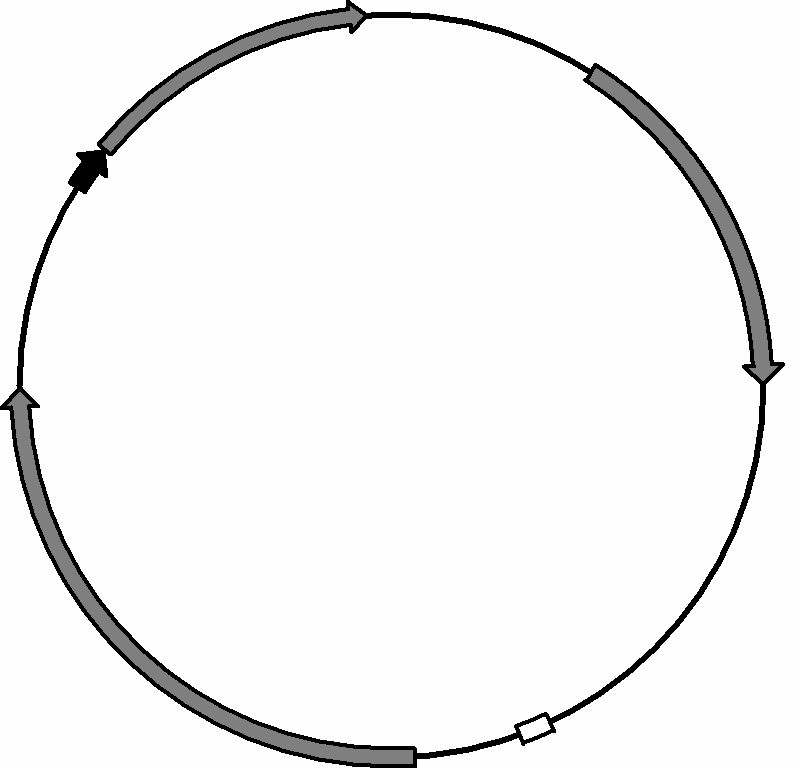


GST

Ptac

LacI^q^

pBR322ori

Amp^r^

pGEX-6P-1-DrTx(1-42)

(5114 bp)

**Figure S1.** Expression vector map showing the position of DrTx(1-42).
