# Supplementary material for: Mutational Analysis of the Analgesic Peptide DrTx(1-42) Revealing a Functional Role of the Amino-Terminal Turn
Source: PLoS One. 2012 Feb 15;7(2):e31830. doi: 10.1371/journal.pone.0031830 (PMC3280213; doi:10.1371/journal.pone.0031830)
Supplement: Figure S2 — Inverse PCR-mediated strategy to construct expression vectors, in which pGEX-6P-1-drosotoxin was initially used as template to generate the DrTx(1-42) expression vector which was further used to construct all the five mutants, including delN (SDGC deleted), D8K, D8A, G9A, and G9R. (DOC) [file pone.0031830.s002.doc]

**Figure S2**

DrTx(1-42)-RP

**• pGEX-6P-1-Drosotoxin**

DrTx(1-42)-FP

*Bam* HI-DDDDK-DGLSGRSDGCYKGPCAVWDNETCRRVCKEEGRSSGHCSPSLKCWCEGLPDNE

KWKYESNTCGS-end-*Sal* I

Inverse PCR

**• pGEX-6P-1-DrTx(1-42)**

delN-FP

delN-RP

*Bam* HI-DDDDK-DGLSGRSDGCYKGPCAVWDNETCRRVCKEEGRSSGHCSPSLK-end-*Sal* I

**• pGEX-6P-1-delN**

Inverse PCR

*Bam* HI-DDDDK-DGLSGR----YKGPCAVWDNETCRRVCKEEGRSSGHCSPSLK-end-*Sal* I

**Figure S2.** Inverse PCR-mediated strategy to construct expression vectors, in which pGEX-6P-1-drosotoxin was initially used as template to generate the DrTx(1-42) expression vector which was further used to construct all the five mutants, including delN (SDGC deleted), D8K, D8A, G9A, and G9R.
