# Supplementary material for: Mutational Analysis of the Analgesic Peptide DrTx(1-42) Revealing a Functional Role of the Amino-Terminal Turn
Source: PLoS One. 2012 Feb 15;7(2):e31830. doi: 10.1371/journal.pone.0031830 (PMC3280213; doi:10.1371/journal.pone.0031830)
Supplement: Figure S3 — RP-HPLC showing the purification of DrTx(1-42). Elution was carried out using a linear gradient of 0–60% acetonitrile in 0.1% trifluoroacetic acid in water (v/v) within 40 min with a flow rate of 1 ml/min. The re-purification is provided in inset. (DOC) [file pone.0031830.s003.doc]

Absorbance (mAU)

DrTx(1-42)

GST

Elution time ( min )

**Figure S3.** RP-HPLC showing the purification of DrTx(1-42). All the elution was carried out using a linear gradient of 0-60% acetonitrile in 0.1% trifluoroacetic acid in water (v/v) within 40 min with a flow rate of 1 ml/min. The re-purification is provided in inset
