# Supplementary material for: Mutational Analysis of the Analgesic Peptide DrTx(1-42) Revealing a Functional Role of the Amino-Terminal Turn
Source: PLoS One. 2012 Feb 15;7(2):e31830. doi: 10.1371/journal.pone.0031830 (PMC3280213; doi:10.1371/journal.pone.0031830)
Supplement: Figure S4 — Determination of molecular weights of HPLC-purified DrTx(1-42) and its mutants by MALDI-TOF. (DOC) [file pone.0031830.s004.doc]

D8A

G9R

G9A

delN

DrTx(1-42)

D8K

**Figure S4.** Determination of molecular weights of HPLC-purified DrTx(1-42) and its mutants by MALDI-TOF.
