# Supplementary material for: Mutational Analysis of the Analgesic Peptide DrTx(1-42) Revealing a Functional Role of the Amino-Terminal Turn
Source: PLoS One. 2012 Feb 15;7(2):e31830. doi: 10.1371/journal.pone.0031830 (PMC3280213; doi:10.1371/journal.pone.0031830)
Supplement: Figure S5 — Time - effect curves of mutants on TTX-R sodium peak currents. The channels were depolarized to −10 mV from a holding potential of −80 mV. A. 8 µM DrTx(1-42); B. 5 µM D8K; C. 30 µM G9R. (DOC) [file pone.0031830.s005.doc]

**A**


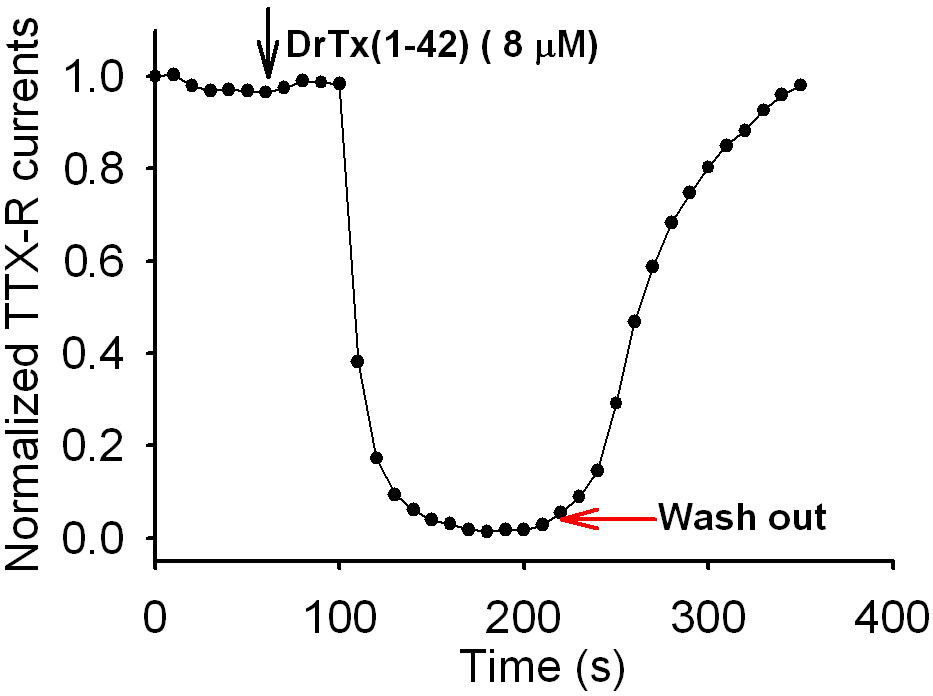


**B**


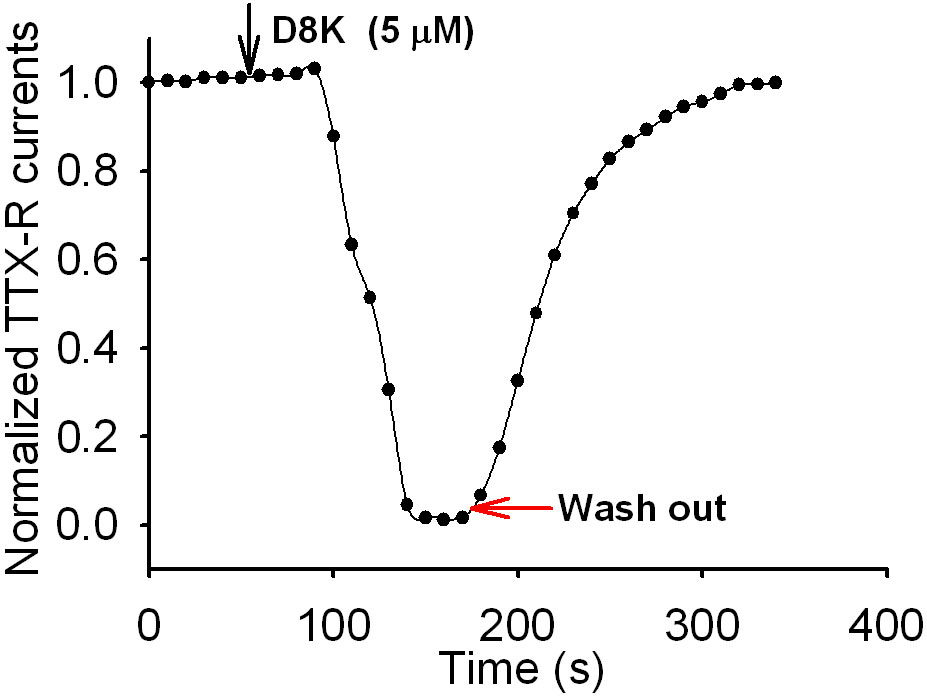


**C**


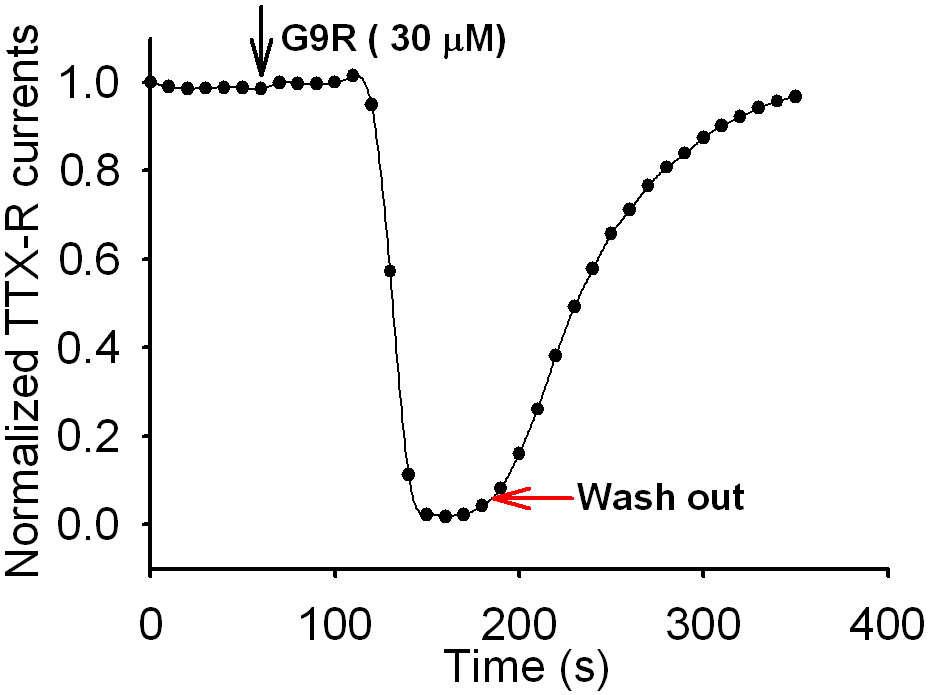


**Figure S5.** Time - effect curves of mutants on TTX-R sodium peak currents. The channels were depolarized to -10 mV from a holding potential of - 80 mV. **A.** 8 μM DrTx(1-42); **B.** 5 μM D8K; **C.** 30 μM G9R.
