# Supplementary material for: Mutational Analysis of the Analgesic Peptide DrTx(1-42) Revealing a Functional Role of the Amino-Terminal Turn
Source: PLoS One. 2012 Feb 15;7(2):e31830. doi: 10.1371/journal.pone.0031830 (PMC3280213; doi:10.1371/journal.pone.0031830)
Supplement: Table S1 — Molecular weights of DrTx(1-42) and its mutants. (DOC) [file pone.0031830.s006.doc]

**Table S1** Molecular weights of DrTx(1-42) and its mutants

| Name | Theoretical (Da) | Experimental (Da) |
| --- | --- | --- |
| DrTx(1-42) | 4528.01 | 4527.20 |
| D8K | 4545.10 | 4544.02 |
| G9R | 4627.15 | 4627.00 |
| D8A | 4484.00 | 4483.82 |
| G9A | 4542.04 | 4541.35 |
| delN | 4167.65 | 4167.56 |
